# Effect of Environmental Noise, Distance and Warning Sound on Pedestrians’ Auditory Detectability of Electric Vehicles

**DOI:** 10.3390/ijerph18179290

**Published:** 2021-09-02

**Authors:** Min-Chih Hsieh, Hung-Jen Chen, Ming-Le Tong, Cheng-Wu Yan

**Affiliations:** 1 Department of Industrial Engineering, University of Shanghai for Science and Technology, No. 516, Jungong Road, Shanghai 201100, China; g9674019@cycu.org.tw (M.-C.H.); gracetong030@gmail.com (M.-L.T.); g9302405@gmail.com (C.-W.Y.); 2 Department of Data Science, Soochow University, No.70, Linhsi Road, Shihlin District, Taipei 111, Taiwan

**Keywords:** electric vehicles, warning sound, environmental noise, auditory detectability, auditory threshold

## Abstract

With developments in science and technology, the number of electric vehicles will increase, and they will even replace ICE vehicles. Thus, perceiving the presence of approaching electric vehicles on the road has become an important issue. In this study, the auditory detectability of the electric vehicle warning sound at different volumes, distances, and environmental noise levels was investigated. To this end, the detection rate was recorded in experiments with three environmental noise levels (50, 60, and 70 dBA), two sound pressure levels (SPLs) of the warning sound (46 and 51 dBA), three frequency combinations of the warning sound (5000, 2500, 1250, and 630 Hz for high frequencies; 2500, 1250, 630, and 315 Hz for medium frequencies; and 1250, 630, 315, and 160 Hz for low frequencies), and five distances (2, 4, 6, 8, and 10 m). The main results showed that the detection rate at 51 dBA was significantly higher than that at 46 dBA under a high-frequency warning sound; however, the detection rates were similar under medium- and low-frequency warning sounds. The participants’ rates of detection for warning sounds were less than 20% under all experimental conditions, and a high-frequency warning sound was not affected by environmental noise. With regard to distances, no significant effects were observed between the distances and the detection rate at any of the three frequencies. In addition, auditory thresholds based on high-, medium-, and low-frequency warning sounds were found through logistic regression analysis results. The results of this study can be used as a reference for the future design of warning sounds.

## 1. Introduction

In the near future, energy sources such as fossil fuels will inevitably become scarce, and therefore, efforts are being made globally to reduce their use. Further, these energy sources cause environmental pollution and damage, and therefore, the prevention and control of environmental pollution, and air pollution in particular, has become an important issue [1]. For instance, the US Environmental Protection Agency amended the Clean Air Act in 1991 to encourage car manufacturers to produce alternative energy vehicles to replace conventional internal combustion engine (ICE) vehicles. The People’s Republic of China (PRC) enacted the Law on the Prevention and Control of Atmospheric Pollution to reduce air pollution by limiting the number of ICE vehicles and encouraging people to buy alternative energy vehicles. The Taiwan Environmental Protection Administration amended the Air Pollution Control Act to improve air quality by speeding up the elimination of old ICE vehicles and banning their sale from 2040.

Owing to the promotion of environmental protection policies and developments in science and technology, the number of electric vehicles (including hybrids) driving legally on the road is expected to increase annually [2]. In addition, although the safety issue is one of the concerns in purchasing electric vehicles [3], the economic benefits [4], driving experience [5], personal environmental awareness [6], pleasure of driving [7], etc., are also reasons influencing the number of electric vehicles. However, even if electric vehicles can contribute to reducing air pollution, they pose risks to pedestrian safety. In the past, pedestrians could judge whether a vehicle was approaching or still distant based on the noise it produced [2,8]. However, previous studies such as Cocron and Krems [9], Fleury et al. [10] and Pardo-Ferreira et al. [11] indicated that electric vehicles generate lower noise levels than ICE vehicles at low speeds, making it more difficult for pedestrians to detect their approach and thereby negatively affecting pedestrian safety [12,13]. Robart and Rosenblum [14] asked participants to estimate the direction of electric vehicles and ICE vehicles moving at 8 km/h through headphones. They found that participants needed more time to understand the direction of electric vehicles than that of ICE vehicles. Electric vehicles are more likely to cause pedestrian traffic accidents than ICE vehicles [15,16]. Therefore, increasing the detection rate of electric vehicles on the road and accordingly improving pedestrian safety are important issues and also the main purpose of this study.

Previous studies have explored the interaction between electric vehicles (including hybrid vehicles in electric mode) and pedestrians and have confirmed that electric vehicles pose risks to pedestrians. Kim et al. [12] recorded the distance at which pedestrians perceived three different types of vehicles approaching at 15 km/h: ICE vehicles, hybrid vehicles in electric mode with added sound, and hybrid vehicles in electric mode without added sound. They found that hybrid vehicles in electric mode without added sound posed a greater risk to pedestrians because their detection distance was shorter than that of ICE vehicles. To increase pedestrian safety, the Acoustic Vehicle Alerting System (AVAS) has been used to increase the audibility of electric vehicles and decrease the incidence of traffic accidents involving pedestrians and electric vehicles. Fleury et al. [10] tested an electric vehicle with four sound modes—no sound, small pitch shift, large pitch shift, and small pitch shift with modulated sine waves—to analyze the effect of adding external sounds on the detection distance. They found that the addition of external sounds resulted in a larger detection distance (i.e., earlier detection), and that modulating the frequency of these sounds resulted in more efficient detection. These study results indicate that electric vehicles with warning sounds are more audible and can thus be detected more easily by pedestrians, increasing road safety.

In addition to the warning sound, other factors that could affect pedestrians’ detection of electric vehicles [17] include pavement and background noise [18]. For example, Mendonça et al. [8] investigated the detection rate of approaching vehicles with different types of pavements (cobble stones, dense asphalt, and open graded), vehicles (small passenger car, hybrid, and pickup truck), and background noises (62, 67, 72, 77, and 82 dBA); they found that all three factors can significantly affect vehicle detection. In particular, the combination of a low-noise pavement and electric vehicles (including hybrid vehicles) may pose the greatest risk to pedestrian safety. Further, Grosse et al. [19] investigated the audibility of ICE vehicles and electric vehicles under two types of background noises: traffic noise and pink noise. They found that even an ICE vehicle is not always audible when the background noise level of both types of noises is 67 dBA. Furthermore, background noise levels vary depending on the environment, thereby influencing the detectability of electric vehicles [20]. For example, Poveda-Martínez et al. [21] conducted detection experiments in three different environments with background noise: three-lane road with various idling vehicles (65.4 dBA), crowded pedestrian street in a shopping area (64.3 dBA), and a park with an equivalent sound (55.3 dBA). They found that different background noises can significantly affect pedestrians’ detection of warning sounds from electric vehicles. From the above studies, we can understand that background noise is one of the important factors that can influence the ability of pedestrians to detect approaching electric vehicles. Thus, to discuss the audibility of warning sounds for electric vehicles, it was necessary to consider the impact of background noises in this study.

Among the above mentioned studies, Grosse et al. [19] used only one sound pressure level (SPL) in their experiment, and Poveda-Martínez et al. [21] recorded background noise in three different environments but used only one SPL to represent the background noise in each environment. However, the SPL of environmental noise varies. For example, traffic noise is greater in peak hours than in off-peak hours and greater near traffic hubs than in residential areas. Nonetheless, some previous studies used background noise existing in the experimental environment as a noise source and seldom considered the fact that the same type of noise would have different SPLs in different environments. Therefore, their results might overestimate or underestimate the audibility and detectability of electric vehicles. In addition, some previous studies conducted experiments with real vehicles in outdoor environments [10,13] or recorded vehicle sounds and played them in the laboratory through headphones or speakers [2,8] to evaluate the audibility and detectability of electric vehicle warning sounds. In these studies, participants heard not only warning sounds but also the noise from tires and pavements. However, different types of tires and pavements will produce different noises, thereby influencing the audibility and detectability of electric vehicles [8,10]. Further, most previous studies recorded the detection distance between the electric vehicle and the participant to evaluate the audibility and detectability of the warning sound of the vehicle. However, an electric vehicle driving on low-noise pavement or equipped with low-noise tires will pose a risk to pedestrians. Thus, when the warning sound, tire type, pavement type, and detection distance are considered together, the results might underestimate the SPL of the warning sound and overestimate the detection distance. Therefore, the effects of the warning sound and detection distance on the audibility and detectability of electric vehicles should be explored without the interference of tire and pavement noise.

In light of the studies cited above, the main objective of the present study was to investigate the influence of the SPL of environmental noise and distance on the audibility and detectability of the warning sound of electric vehicles. Further, the relationship among the warning sound, environmental noise, and distance was formulated in this study.

## 2. Material and Methods

### 2.1. Participants

Thirty-three participants were recruited and participated voluntarily in this study. All participants were informed that normal hearing was a requirement for the test. The frequency of the normal hearing test was set between 500 and 4000 Hz as measured using an audiometer [10]. Three participants were excluded owing to hearing problems or because they misunderstood the test instructions. Finally, 30 participants (9 females and 21 males, ages: 18–24 years, M = 20, SD = 4.59) performed in the experiments. All participants were university students from University of Shanghai for Science and Technology. This study was submitted to the Research Ethics Committee of University of Shanghai for Science and Technology on 1 April 2020, and ethical review and approval were waived for this study, due to the fact that the data collection involved no greater than minimal risk to participants and the data collected did not contain personally identifiable information on any individual participant.

### 2.2. Environmental Noise

Environmental noises play an important role in the auditory detectability of electric vehicles. Thus, this study evaluated the audibility and detectability of the warning sounds of electric vehicles under environmental noises with different SPLs.

**Figure 1:**
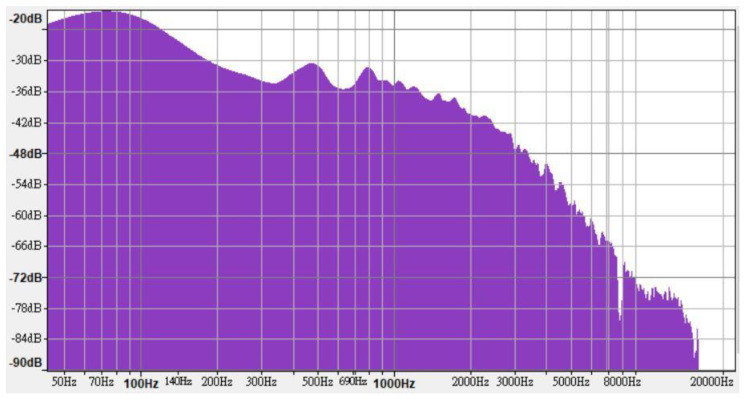
Frequency characteristics of traffic noise sample.

Road traffic is the most common and important source of environmental noise [21]. Thus, traffic noise was used as environmental noise in this experiment. Noise samples were recorded at the eight-lane T-shaped intersection of Jungong road and Zhoujiazui road, Shanghai, using a Philips VTR9200 at 4-m distance from the reflector (i.e., building walls) and 1.65-m height using the measurement methodology described in the national standards of the PRC (document no. GB 3096-2008). The noise samples were recorded in the peak period of 9:00–9:15 AM on a sunny morning. As a result, the noise samples contained the sounds of bicycles, motorcycles, cars, buses, and trucks, thereby better simulating the sounds that pedestrians may hear when walking on the road. Figure 1 shows the frequency characteristics of the traffic noise sample.

### 2.3. Stimuli

The warning sounds of electric vehicles must be synthesized so as to avoid the influence of the tire and pavement noise on their identification. Bodies such as the United Nations, US National Highway Traffic Safety Administration, and the Standardization Administration of the PRC have established regulations on the characteristics of warning sounds. This study was conducted in Shanghai; therefore, the warning sounds used in the experiment were synthesized following the national standards of the PRC (document no. GB/T 37153-2018). This regulation states that when warning sounds are synthesized, their frequencies must include at least two one-third octave bands, of which at least one must be below 1.6 kHz.

**Figure 2:**
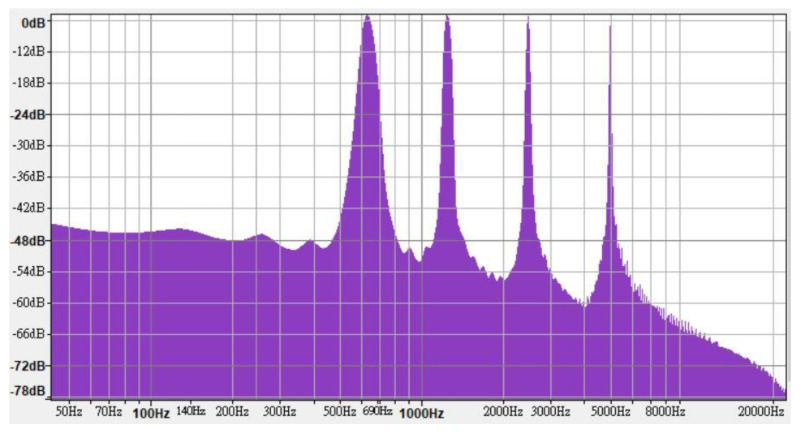
Frequency characteristics of high-frequency warning sound.

**Figure 3:**
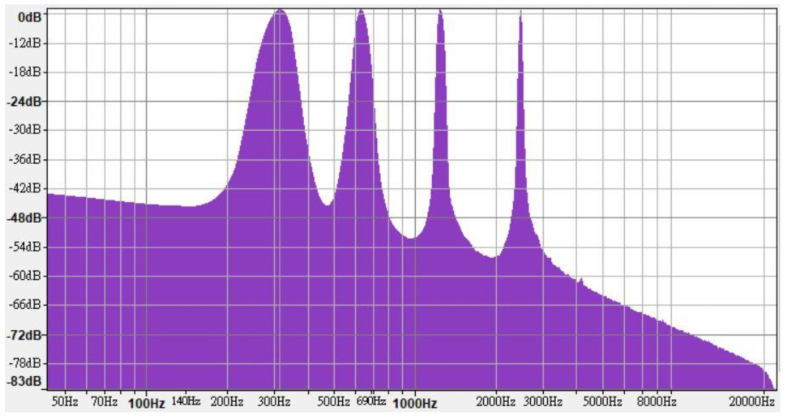
Frequency characteristics of medium-frequency warning sound.

**Figure 4:**
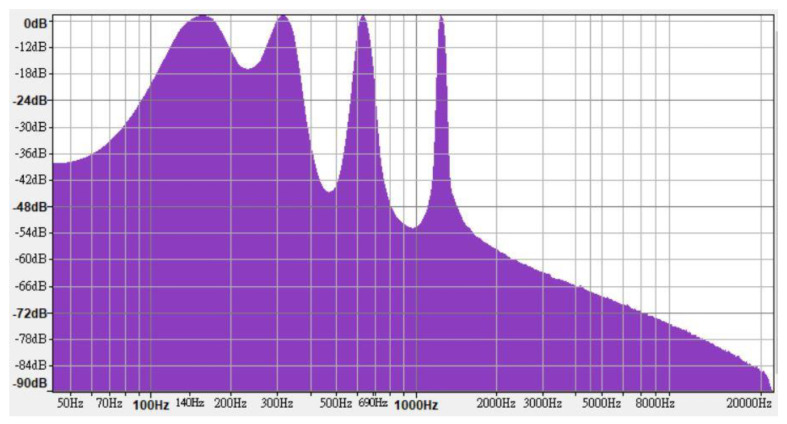
Frequency characteristics of low-frequency warning sound.

In line with the regulations, high-, medium-, and low-frequency warning sounds were synthesized using FL Studio 12; each frequency range contained four one-third octave bands. The high-, medium-, and low-frequency warning sounds contained frequencies of 5000, 2500, 1250, and 630 Hz (Figure 2); 2500, 1250, 630, and 315 Hz (Figure 3); and 1250, 630, 315, and 160 Hz (Figure 4), respectively.

### 2.4. Experimental Design

To simulate environmental noise with different SPLs, the upper limits for noise in the environment and functional areas specified in the environmental quality standard for noise (document no. GB 3096-2008) were used as a reference in this experiment. The upper limit of daytime environmental noise is 50 dBA in rehabilitation and convalescent areas; 60 dBA in residential, commercial, and industrial mixed areas; and 70 dBA along the sides of highways, urban expressways, urban trunk roads, etc. Therefore, 50, 60, and 70 dBA were used as the SPLs of environmental noise in this experiment.

When the vehicle speed exceeds 10 and 20 km/h, the SPL of the warning sound must not be lower than the minimum levels of 46 and 51 dBA, respectively, as specified in GB/T 37153-2018. These SPL values were used in this experiment.

This study also investigated the detection distance between the warning sound and the participants. This distance might have been overestimated in previous studies. Thus, the detection distance between the warning sounds and the participants was selected as an independent variable and investigated in relation to the environmental noise, warning sound, and distance. Owing to the lack of a reference value, this study used 2, 4, 6, 8, and 10 m as distances between the warning sound and the participants to evaluate the detection rate of the warning sound at different distances.

This study evaluated four independent variables: SPL of environmental noise (50, 60, and 70 dBA), SPL of warning sound (46 and 51 dBA), frequency of warning sound (5000, 2500, 1250, and 630 Hz for high frequencies; 2500, 1250, 630, and 315 Hz for medium frequencies; and 1250, 630, 315, and 160 Hz for low frequencies), and distance between warning sound and participant (10, 8, 6, 4, and 2 m). Therefore, a balanced factorial design with 3 (SPL of environmental noise) × 2 (SPL of warning sound) × 3 (frequency of warning sound) × 5 (distance) combinations (i.e., 90 combinations) was obtained. All factors were treated as within-subject factors.

### 2.5. Experimental Process

**Figure 5:**
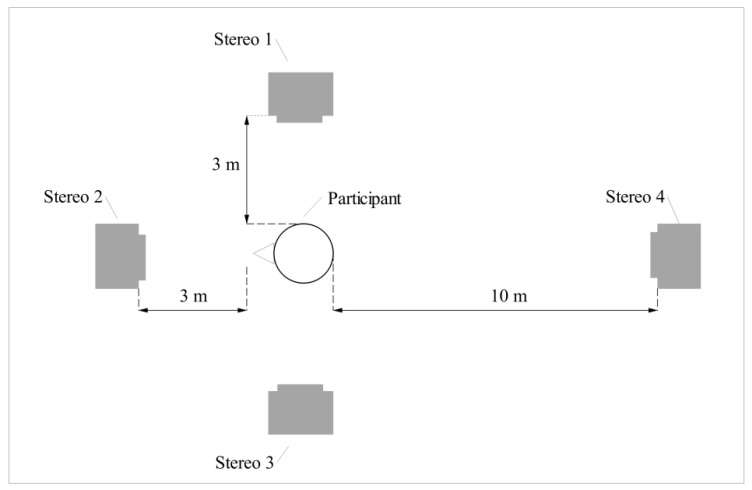
Experimental setting. Stereos 1–3 were used to play environmental noise, and stereo 4 was used to play warning sounds. The distance of stereo 4 from the participant could be changed according to the experimental conditions.

Experiments were conducted in the outdoor multifunctional stadium of Shanghai University for Science and Technology. To play ambient noise and warning sounds, four Philips SD60S hi-fi Bluetooth stereos were used. Of these, three were used as sound sources of environmental noise and one was used as the sound source of the synthesized warning sounds. Figure 5 shows the experimental setting.

Normal hearing tests were conducted with each participant to ensure that they could correctly identify the warning sounds. Once participants passed this test, they were allowed to perform in the formal experiment. To enable participants to identify the warning sound more accurately, a 10-s warning sound was played before the formal experiment. Then, participants were asked to identify the warning sound as soon and as often as possible during the formal experiment.

**Figure 6:**
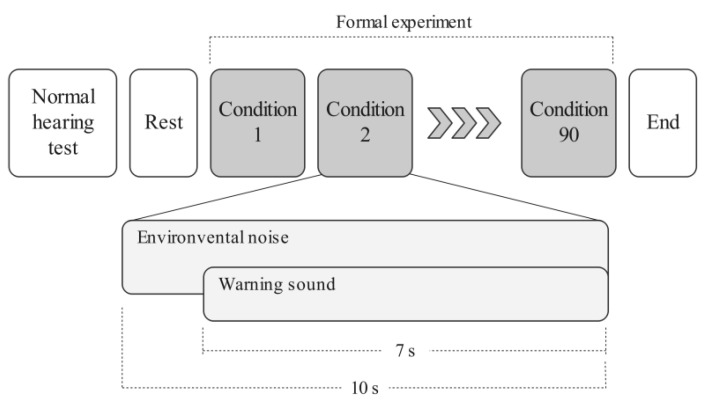
The experimental process in this study.

According to the experimental design, each participant was required to perform in all 90 combinations of experimental conditions, and these conditions were tested in a randomized order. In the formal experiment, each participant was asked to sit in a chair with their back facing the source of the warning sound. Environmental noise alone was played for 3 s, and then the warning sound was played alongside the environmental noise for a further 7 s; in other words, the noise and sound were played for 10 s. During this period, participants only had to answer whether or not they had heard the warning sound. Once a participant answered this question, the next experimental condition was tested. The experimental process of this study is shown in Figure 6.

### 2.6. Statistical Analysis

Experimental data for high-, medium-, and low-frequency warning sounds were analyzed. The Kruskal–Wallis test was applied to evaluate the influence of the SPL of the warning sound, SPL of environmental noise, and distance on the detection rate.

Because the experimental data were binary in nature, a logistic regression was applied to identify the relationships among the SPL of the warning sound, SPL of environmental noise, distance, and detection rate under high-, medium-, and low-frequency warning sounds.

## 3. Results

### 3.1. Detection Rate

Overall, 90 combinations of the SPL of the warning sound, SPL of environmental noise, distance, and frequency were tested in the experiments. Table 1 shows the Kruskal–Wallis test results for the effects of these combinations on the detection rate. The results indicated that the SPLs of the warning sound and environmental noise significantly affected the detection rate. With regard to the SPL of the warning sound, a post hoc analysis indicated that the detection rate at 51 dBA (11.26%) was significantly higher than that at 46 dBA (6.96%) (*χ*^2^ = 9.742, *p* < 0.05). With regard to the SPL of environmental noise, a post hoc analysis indicated that the warning sound detection rates at 70 and 60 dBA (6.78% and 7.56%, respectively) were significantly lower than that at 50 dBA (13%).

Table 2 shows the Kruskal–Wallis test results for the effects of the three frequencies of warning sounds on the detection rate. The SPL of the warning sound significantly affected the detection rate for a high-frequency warning sound (*χ*^2^ = 7.523, *p* < 0.05), and the SPL of environmental noise significantly affected the detection rate for medium- (*χ*^2^ = 16.447, *p* < 0.05) and low-frequency (*χ*^2^ = 8.629, *p* < 0.05) warning sounds. However, the distance did not affect the detection rate for all three frequencies.

**Figure 7:**
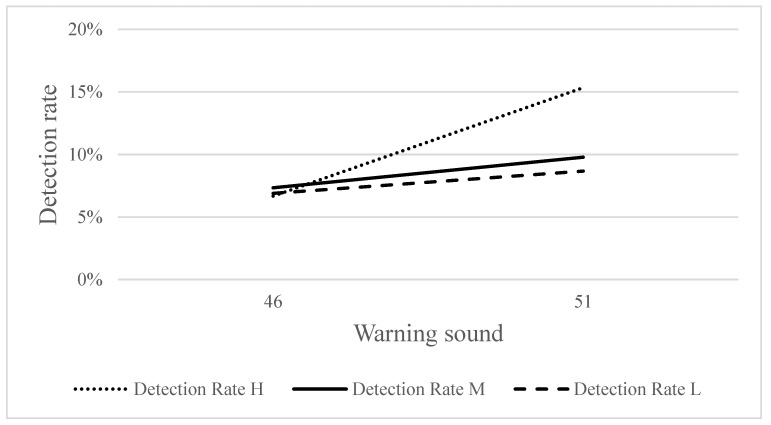
Detection rate with two SPLs of warning sounds under three frequencies of warning sounds (H, M, and L indicate high-, medium-, and low-frequency warning sounds).

Figure 7 shows the detection rates for the three frequencies of warning sounds under two SPLs of warning sounds. The Kruskal–Wallis test results indicated that the detection rate at 51 dBA (15.33%) was significantly higher than that at 46 dBA (6.67%) (*χ*^2^ = 7.523, *p* < 0.05) under a high-frequency warning sound; however, the detection rates were similar under medium- and low-frequency warning sounds.

**Figure 8:**
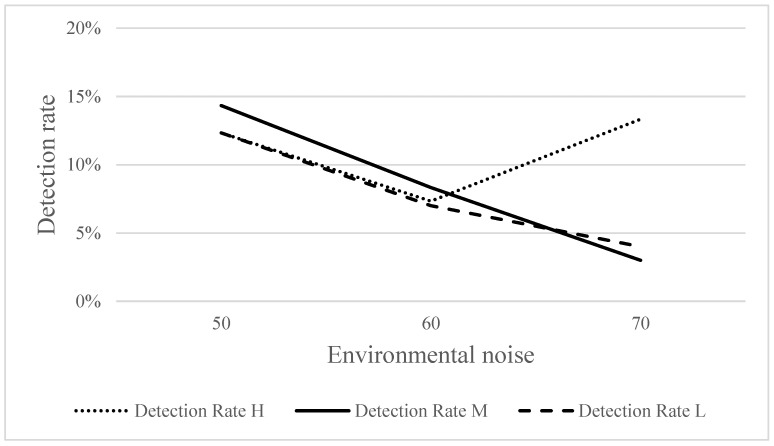
Detection rates for three frequencies of warning sounds under different SPLs of environmental noise.

Figure 8 shows the detection rates for the three frequencies of warning sounds under three SPLs of environmental noise. A post hoc analysis showed that the detection rate at 70 dBA (3%) was significantly lower than that at 50 dBA (14.33%) (*χ*^2^ = 15.750, *p* < 0.05) for medium-frequency warning sound, and that at 70 dBA (4%), it was significantly lower than that at 50 dBA (12.33%) (*χ*^2^ = 11.300, *p* < 0.05) for the low-frequency warning sound. However, no significant difference was observed for the high-frequency warning sound.

**Figure 9:**
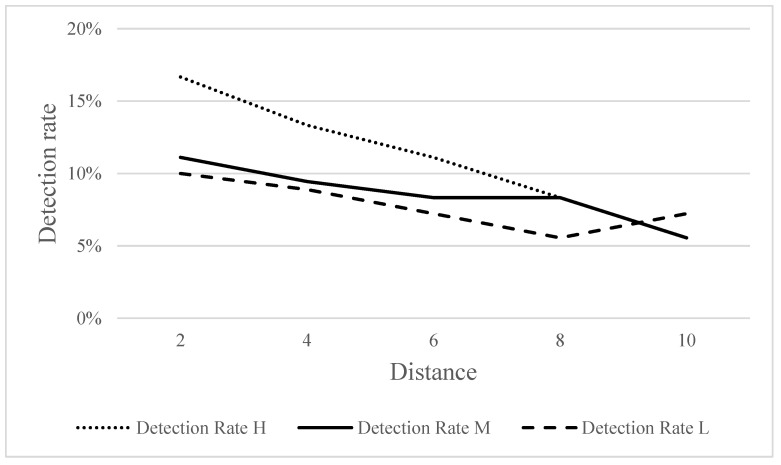
Detection rates for three frequencies of warning sounds at different distances.

Figure 9 shows the detection rates of the three frequencies of warning sounds at different distances. No significant effects were observed between distance and detection rate at all three frequencies.

### 3.2. Logistic Regression Analysis

Logistic regression analyses were performed to identify the relationships between the SPL of warning sound, SPL of environmental noise, distance, and detection rate. The dependent variable, namely, the detection rate, was divided into three groups based on the frequency; specifically, P_H_, P_M_, and P_L_—representing the detection rates for high-, medium-, and low-frequency warning sounds, respectively. The independent variables were the SPL of warning sound (X_WS_), SPL of environmental noise (X_EN_), and distance (X_D_). The following equations were obtained:

(1) $$\mathrm{Ln}\left(\frac{P_{H}}{1-P_{H}}\right)=-10.847+0.189X_{\mathrm{WS}}+0.005X_{\mathrm{EN}}-0.146X_{D}$$

(2) $$\mathrm{Ln}\left(\frac{P_{M}}{1-P_{M}}\right)=-0.491+0.065X_{\mathrm{WS}}-0.079X_{\mathrm{EN}}-0.081X_{D}$$

(3) $$\mathrm{Ln}\left(\frac{P_{L}}{1-P_{L}}\right)=-0.995+0.051X_{\mathrm{WS}}-0.061X_{\mathrm{EN}}-0.063X_{D}$$

These equations can be rewritten as follows:

(4) $$P_{H}=\frac{e^{-10.847+0.189X_{\mathrm{WS}}+0.005X_{\mathrm{EN}}-0.146X_{D}}}{e^{-10.847+0.189X_{\mathrm{WS}}+0.005X_{\mathrm{EN}}-0.146X_{D}}+1}$$

(5) $$P_{M}=\frac{e^{-0.491+0.065X_{\mathrm{WS}}-0.079X_{\mathrm{EN}}-0.081X_{D}}}{e^{-0.491+0.065X_{\mathrm{WS}}-0.079X_{\mathrm{EN}}-0.081X_{D}}+1}$$

(6) $$P_{L}=\frac{e^{-0.995+0.051X_{\mathrm{WS}}-0.061X_{\mathrm{EN}}-0.063X_{D}}}{e^{-0.995+0.051X_{\mathrm{WS}}-0.061X_{\mathrm{EN}}-0.063X_{D}}+1}$$

**Figure 10:**
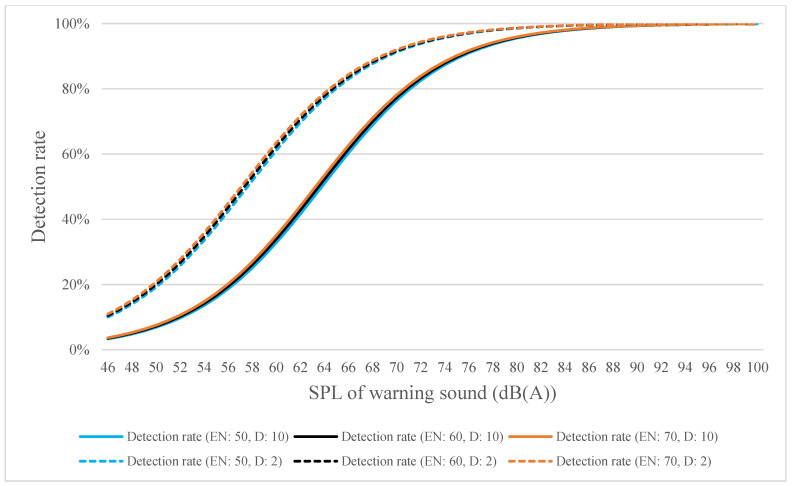
Estimated detection rate for high-frequency warning sound as calculated using Equation (4) (EN denotes environmental noise; D denotes distance).

**Figure 11:**
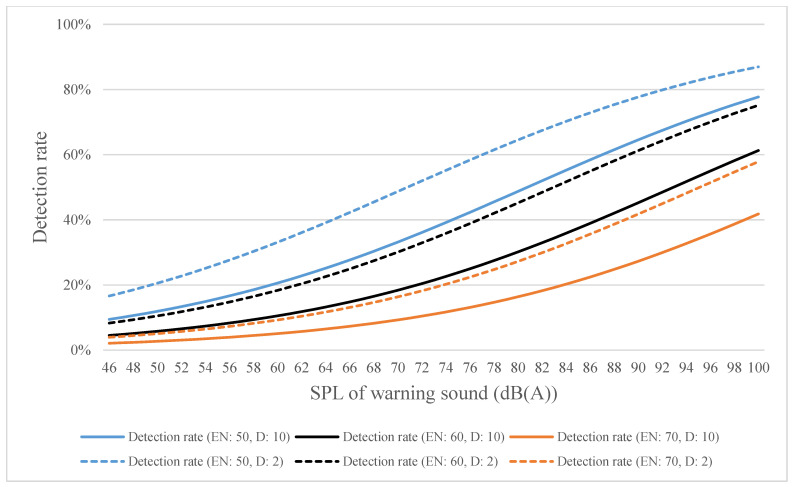
Estimated detection rate for medium-frequency warning sound as calculated using Equation (5) (EN denotes environmental noise; D denotes distance).

**Figure 12:**
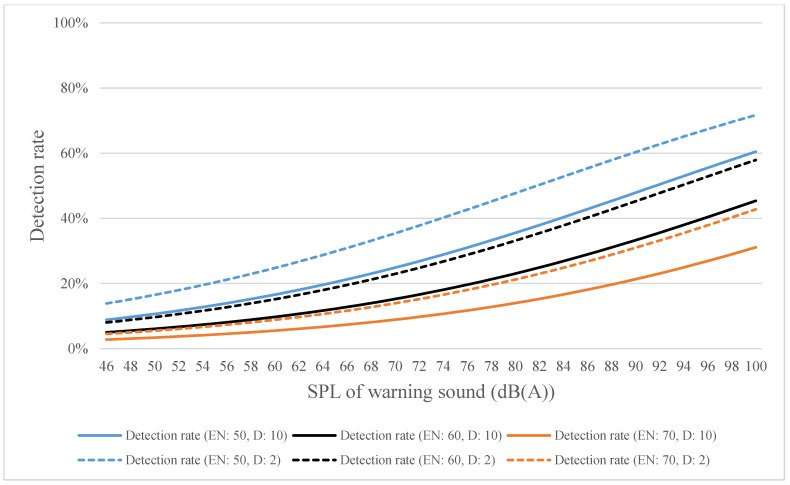
Estimated detection rate for low-frequency warning sound as calculated using Equation (6) (EN denotes environmental noise; D denotes distance).

By using distances of 2 and 10 m as an example, the estimated detection rates for high-, medium-, and low-frequency warning sounds were calculated using Equations (4)–(6) and plotted in Figure 10, Figure 11 and Figure 12, respectively. Figure 10 shows that when the SPL of the warning sound was increased by 1 dB, the detection rate increased by 1.208 times; when the SPL of environmental noise increased by 1 dB, the detection rate increased by 1.005 times; and when the distance increased by 1 m, the detection rate increased by 0.864 times. Figure 11 shows that when the SPL of the warning sound increased by 1 dB, the detection rate increased by 1.067 times; when the SPL of environmental noise increased by 1 dB, the detection rate increased by 0.924 times; and when the distance increased by 1 m, the detection rate increased by 0.922 times. Figure 12 shows that when the SPL of the warning sound increased by 1 dB, the detection rate increased by 1.052 times; when the SPL of environmental noise increased by 1 dB, the detection rate increased by 0.940 times; and when the distance increased by 1 m, the detection rate increased by 0.939 times. The logistic regression analysis indicates that the precision of the estimated detection rates for high-, medium-, and low-frequency warning sounds was 89%, 91.4%, and 92.2%, respectively. In addition, the Hosmer–Lemeshow test was used to test the goodness-of-fit (GOF) of the equations in this study, and the results of the GOF test show that Equations (1)–(3) fit the observed data well (the *p*-values for Equations (1)–(3) were 0.665, 0.452, and 0.727, respectively).

Determining whether a pedestrian can detect an electric vehicle approaching from behind can be considered analogous to determining the absolute threshold of target detection. Previous studies [22,23] typically defined the threshold as the lowest intensity that a person can detect 50% of the time. Considering a medium-frequency warning sound as an example, when using a 50% detection rate for distances of 10 and 2 m and environmental noise of 50 dBA, the estimated SPLs of the warning sound as calculated using Equation (5) were ~81 dBA and 71 dBA, respectively. Table 3, Table 4 and Table 5 respectively show the estimated SPLs of high-, medium-, and low-frequency warning sounds that are detectable by pedestrians with certain detection rates.

## 4. Discussion

In general, the results of this study showed that the participants had a higher detection rate for high-frequency warning sounds than for medium- and low-frequency warning sounds. As regards the SPL of warning sounds, the detection rate of high-frequency warning sounds was significantly higher at 51 dBA than at 46 dBA, whereas no significant differences were seen for medium- and low-frequency warning sounds. Logistic regression analysis showed that when the SPL of the warning sound increased by 1 dB, the detection rate of a high-frequency warning sound increased by a factor of 1.208 whereas those of medium- and low-frequency warning sounds increased only by factors of 1.067 and 1.052, respectively. Therefore, subjects may be more sensitive to high-frequency warning sounds than to medium- and low-frequency warning sounds.

### 4.1. Environmental Noise

Further, the detection rate of high-frequency warning sounds seemed unaffected by environmental noise. No significant difference in detection rate was observed between different SPLs of environmental noise for high-frequency warning sounds, whereas medium- and low-frequency warning sounds were affected by environmental noise (i.e., masking effect). The louder the environmental noise, the lower was the detection rate. Previous studies have also reported higher detection rates with high-frequency sounds [24]. However, the more similar the environmental noise is to the frequency of the target sound, the more likely it is to interfere with pedestrians’ judgment [10,25]. As long as the dominant frequency bands of the target sound are prominent relative to the ambient noise, the target sound should be unaffected by the masking effect [21]. For the high-frequency warning sound used in this study, the amplitudes of 5000 and 2500 Hz far exceeded those of environmental noise; therefore, this sound was unaffected by environmental noise and was more easily detected by participants than medium- and low-frequency warning sounds.

### 4.2. Distance

No significant differences were seen in the detection rates for high-, medium-, and low-frequency warning sounds at different distances. Although distance did not have a statistically significant impact on participants’ judgment, Figure 9 shows that the detection rate decreased with increasing distance. Therefore, the distance does affect participants’ judgment, but not in an obvious manner within 10 m. In general, the experimental results of this study for environmental noise, warning sound, distance, and detection rate are congruent with generally accepted knowledge in acoustics.

### 4.3. Logistic Regression Analysis

The detection rate was low, being less than 20%, under all experimental conditions, indicating that participants could not easily detect the warning sound. In other words, if the warning sound of electric vehicles is designed in accordance with current regulations, its minimum SPL is likely too low to be detectable by pedestrians, thereby posing a risk to pedestrians’ safety. Logistic regression analysis indicated that participants’ auditory threshold was between 57 dBA (2 m) and 64 dBA (10 m) when using the high-frequency warning sound, and the SPL of this sound needed to reach 84–86 dBA for participants to always hear it; however, this far exceeds environmental quality standards and regulations for noise (document no. GB 3096-2008). Participants’ auditory threshold for both medium- and low-frequency warning sounds was greater than 70 dBA and even exceeded 100 dBA (Table 4 and Table 5, respectively); therefore, these sounds would have to exceed 100 dBA to always be audible. This result indicates that the frequency distribution of medium- and low-frequency warning sounds used in this study was unsuitable for designing warning sounds for electric vehicles. In contrast, the high-frequency warning sound used in this study was unaffected by environmental noise, and its auditory threshold meets environmental noise regulations (document no. GB 3096-2008). Thus, it is more suitable for designing warning sounds for electric vehicles.

### 4.4. Limitations

This study has three limitations. First, its results may not be applicable to different populations. For example, Stelling-Konczak et al. [16] noted increased hearing loss with age, especially for high-frequency noise and for the elderly [26]. However, the participants in this study were aged 18–28 years. Therefore, the applicability of these results to pedestrians of different age groups requires further investigation. Second, many frequency combinations can be used to design warning sounds, of which this study used only some. Whether the results of this study are valid for other frequency combinations remains unknown. Third, the results of this study only addressed whether or not the warning sound could be heard; therefore, they pertained to the perception stage in human information processing (HIP). Participant response time after hearing the warning sound was not included in the scope of this study.

## 5. Conclusions

This study investigated the effects of the SPL and frequency of warning sound, SPL of environmental noise, and distance on the audibility of the warning sound of electric vehicles. To this end, logistic regression analysis was used to identify potential relationships between these factors. The results showed that the detection rate for medium- and low-frequency warning sounds is affected by environmental noise, whereas that of high-frequency warning sound is not. Further, the distance between the sound source and the participants did not affect the detection rate. Additionally, this study found that the detection rate for the warning sound under three environmental noise levels was less than 20%. This may explain why pedestrians suffer traffic accidents with electric vehicles. The relationships between the SPL of warning sound, SPL of environmental noise, distance, and detection rate were integrated into three equations based on high-, medium-, and low-frequency warning sounds, respectively. According to the results of this study, the frequency of the warning sound should be increased and the SPL should not be lower than 57 dB so that pedestrians have at least a 50% chance of hearing the warning sound of electric vehicles and taking appropriate precautions.

In the near future, the number of electric vehicles will increase and replace ICE vehicles. However, the increasing popularity of electric vehicles may make the results of this study inapplicable because this study only considered electric vehicles as a unique presence in the environment. Hence, future study could focus on exploring various factors for designing the warning sounds of electric vehicles or the manner of communication between electric vehicles and pedestrians to improve pedestrian safety.

## Figures and Tables

**Table 1:** Overall Kruskal–Wallis test results for detection rate.

	Chi-Square	*p*-Value
Warning sound	9.742	0.002 *
Environmental noise	22.388	<0.001 *
Distance	6.412	0.170
Frequency	1.750	0.417

*: *p* < 0.05.

**Table 2:** Kruskal–Wallis test results for detection rate with high-, medium-, and low-frequency warning sounds.

	High Frequency		Medium Frequency		Low Frequency	
	Chi-Square	*p*-Value	Chi-Square	*p*-Value	Chi-Square	*p*-Value
Warning sound	7.523	0.006 *	1.810	0.179	2.120	0.145
Environmental noise	2.612	0.271	16.447	<0.001 *	8.629	0.013 *
Distance	4.252	0.373	2.991	0.559	1.525	0.822

*: *p* < 0.05.

**Table 3:** Estimated SPL (dBA) of high-frequency warning sound.

Distance	2 M			10 M		
**Environmental Noise**	**50**	**60**	**70**	**50**	**60**	**70**
**Detection Rate**						
**20%**	50	50	50	56	56	56
**30%**	53	53	53	59	59	59
**40%**	55	55	55	62	61	61
**50%**	**58**	**57**	**57**	**64**	**64**	**63**
**60%**	60	60	59	66	66	65

**Table 4:** Estimated SPL (dBA) of medium-frequency warning sound.

Distance	2 M			10 M		
**Environmental Noise**	**50**	**60**	**70**	**50**	**60**	**70**
**Detection Rate**						
**20%**	50	62	74	59	72	84
**30%**	58	70	82	68	80	92
**40%**	65	77	89	74	87	99
**50%**	**71**	**83**	**95**	**81**	**93**	**>100**
**60%**	77	89	>100	87	99	>100

**Table 5:** Estimated SPL (dBA) of low-frequency warning sound.

Distance	2 M			10 M		
**Environmental Noise**	**50**	**60**	**70**	**50**	**60**	**70**
**Detection Rate**						
**20%**	54	66	78	64	76	89
**30%**	65	77	89	75	87	99
**40%**	74	86	98	84	96	>100
**50%**	**82**	**94**	**>100**	**92**	**>100**	**>100**
**60%**	90	>100	>100	100	>100	>100

## Data Availability

The datasets generated and analyzed during the current study will not be publicly available due to privacy and confidentiality reasons.
